# A Multifunctional and Possible Skin UV Protectant, (3*R*)-5-Hydroxymellein, Produced by an Endolichenic Fungus Isolated from *Parmotrema austrosinense*

**DOI:** 10.3390/molecules22010026

**Published:** 2016-12-26

**Authors:** Lu Zhao, Jin-Cheol Kim, Man-Jeong Paik, Wonjae Lee, Jae-Seoun Hur

**Affiliations:** 1 Korean Lichen Research Institute, Sunchon National University, 255 Jungang-Ro, Suncheon 57922, Korea; zhaolushuijing@163.com; 2 Department of Agricultural Chemistry, Institute of Environmentally Friendly Agriculture, College of Agriculture and Life Sciences, Chonnam National University, 77 Yongbong-ro, Gwangju 500-757, Korea; kjinc@chonnam.ac.kr; 3 College of Pharmacy, Sunchon National University, 255 Jungang-Ro, Suncheon 57922, Korea; paik815@sunchon.ac.kr; 4 College of Pharmacy, Chosun University, Gwangju 61452, Korea; wlee@chosun.ac.kr

**Keywords:** antioxidant, endolichenic fungus, (3*R*)-5-hydroxymellein, melanin, UV protection

## Abstract

Lichens are considered a great bio-resource because they produce large numbers of secondary metabolites with many biological activities; however, they have not been cultivated under artificial conditions to date. As a result, lichen substances from natural sources are limited and have not been widely utilized in commercial applications. Accordingly, interest in lichen-associated fungi, especially endogenic fungi, has increased. Ultraviolet (UV) radiation in sunlight is harmful to human health, resulting in demand for effective UV filtering agents for use in sunscreen. In this study, we purified (3*R*)-5-hydroxymellein, which has UVA absorption activity, from the secondary metabolites of an endolichenic fungus (ELF000039). The antioxidant properties were then assessed by in vitro tests. The antioxidant activity of (3*R*)-5-hydroxymellein was high when compared to the recognized antioxidants ascorbic acid (ASA) and butyl hydroxyl anisole (BHA). Moreover, the compound exhibited no cytotoxicity toward mouse melanoma cell lines, B16F1 and B16F10, or the normal cell line, HaCaT. Furthermore, (3*R*)-5-hydroxymellein recovered the damage caused by UVB irradiation and inhibited melanin synthesis. Taken together, these results suggest that (3*R*)-5-hydroxymellein could have an interesting and vital profile to go further development as a multifunctional skin UV protectant.

## 1. Introduction

Ultraviolet (UV) rays exist in sunlight at wavelengths ranging from 1 nm to 400 nm. UVA (315–400 nm) and UVB (280–315 nm) can pass through the ozone layer and reach the ground, whereas UVC (<280 nm) cannot [1]. It is well known that the long term effects of UV radiation are harmful to human health and that excessive doses can cause photo-aging, inflammation, and malignant skin cancers [2,3,4]. Melanoma is an aggressive skin cancer that has been increasing in humans worldwide, and it is estimated that about 48,000 people die from melanoma annually. The use of sunscreen is the most common and convenient method of protecting the population from UV radiation [5].

UV protectants are a kind of substance that can help protect against the damaging effects of UV radiation. Organic compounds, especially natural products, are generally considered as the primary source of UV protectants due to their UV absorption property. Some of them have been commonly utilized in cosmetics, such as benzophenones, para-aminobenzoic acid (PABA) and cinnamates [6,7].

Endolichenic fungi (ELF) live within the lichen thallus in the same way that endophytes live between cells in plant tissues [8]. Although lichens have been shown to possess a variety of biological activities, few studies have investigated the chemical and biological activity of secondary metabolites of ELF [9]. In the present study, we purified (3*R*)-5-hydoxymellein, which has UVA absorption activity from secondary metabolites of the endolichenic fungus species, ELF000039. Antioxidant (DPPH radical scavenging, reducing power and superoxide anion scavenging activity, and inhibition of linoleic acid peroxidation) properties of (3*R*)-5-hydroxymellein were assessed by in vitro tests. To explore the effects of (3*R*)-5-hydroxymellein on cells, the cytotoxicity of (3*R*)-5-hydroxymellein against the mouse melanoma cell lines, B16F1 and B16F10, and the normal cell line, HaCaT, were determined. Additionally, the ability to recover UVB-induced damage and inhibit melanin synthesis was investigated to corroborate its function as a UV protectant.

It is well known that sunscreens have a large market with increasing demand because existing UV protective ingredients do not satisfy the multiple needs of consumers. On the one hand, the safety problems of existing commercial UV filters were increasingly proved; additionally, people would prefer whitening, anti-aging, and other functions in sunscreens besides UV protective effect; therefore, there is an urgent need to identify multifunction skin UV protectants. In this study, we investigated whether (3*R*)-5-hydroxymellein, a natural product, has UV absorption and the related bioactivities in vitro tests as a potential skin UV protectant.

## 2. Results

### 2.1. UV Absorption Activity of Secondary Metabolites from the Endolichenic Fungus

**Figure 1:**
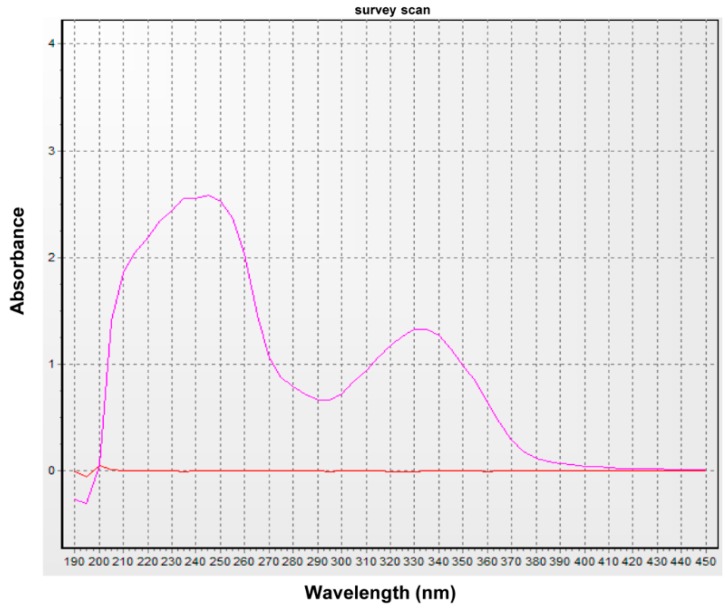
UV spectrum of ELF000039 crude extract after 28 days of culture. The orange line is the UV spectral baseline of the solvent (ethanol).

**Figure 2:**
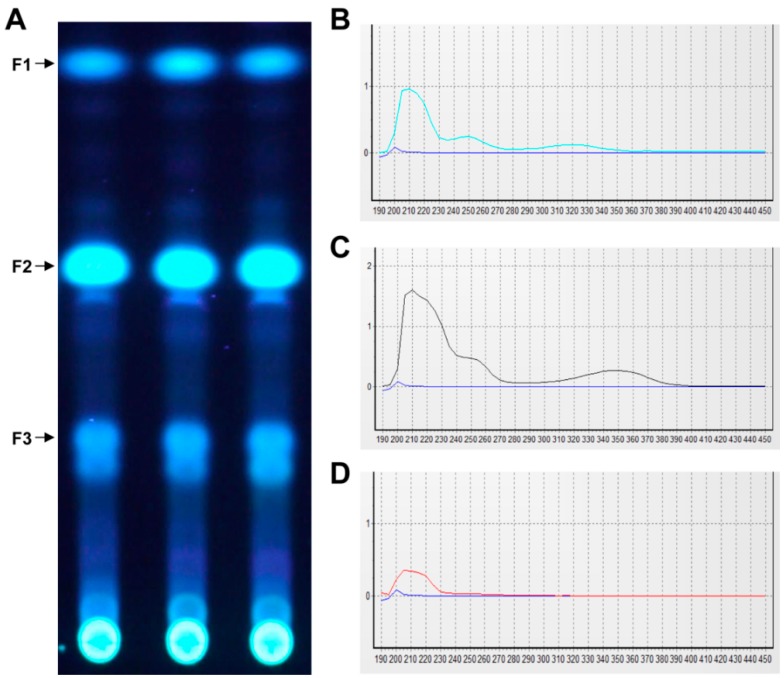
(**A**) TLC analysis of the main fractions of ELF000039 crude extract under UV light; (**B**–**D**) UV spectra of three main fractions (F1, F2, and F3). The blue line is the UV spectral baseline of the solvent.

Analysis of the ITS sequence based on BLAST searches of the GenBank database showed that our fungal strain (GenBank Accession No. KX765309) had very low similarity to known species and could be a new fungal species. Based on the UV spectrum of the crude extract (Figure 1), secondary metabolites contained substances that produced an absorption peak at about 335 nm, which is in the UVA range. To confirm which fraction of the crude extract had UV absorption activity, the three main fractions were obtained by prep-TLC. The UV spectra showed that fraction 2 (F2) produced an absorption peak at 350 nm (Figure 2).

### 2.2. F2 Isolation and Identification

**Figure 3:**
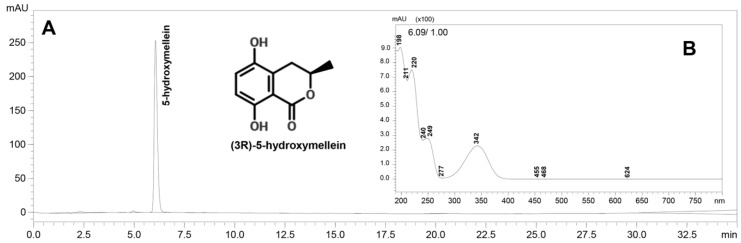
(**A**) HPLC chromatogram of the purified single compound acquired at 254 nm. The molecular structure of (3*R*)-5-hydroxymellein is presented in the insets; (**B**) UV spectra of the purified single compound (0.2 mg/mL).

F2 was isolated and purified via silica gel columns and a Sephadex column, successively. The purity of the isolated compound was confirmed by HPLC analysis. Only one peak (RT = 6.087 min) was observed in the results of the HPLC analysis, and the UV spectra suggested that the maximum absorption wavelengths (λ_max_) of the compounds were 198 nm (ε = 17,476 L·mol^–1^·cm^–1^), 220 nm (ε = 14,564 L·mol^−1^·cm^–1^), 249 nm (ε = 5437 L·mol^–1^·cm^–1^), and 342 nm (ε = 4466 L·mol^–1^·cm^–1^) (Figure 3). The compound absorbed UVA at 342 nm wavelength with a moderate absorption coefficient corresponding to the results of previous UV scanning spectra.

In the electron ionization mass spectrum generated by GC-MS analysis, the molecular ions of the isolated metabolite had an *m*/*z* value of 194, which included the fragmented ions of *m*/*z* 176, 165 and 150. This compound was identified as 5-hydroxymellein based on the GC-MS Willey library.

The ^1^H-NMR spectra exhibited signals at *δ* 7.02 (1H, d, *J* = 9.0 Hz, H-6), δ 6.70 (1H, d, *J* = 9.0 Hz, H-7), δ 3.17 (1H, dd, *J* = 3.5, 16.5 Hz, H-4), δ 2.62 (1H, dd, *J* = 11.5, 16.5 Hz, H-4), and 1.50 (3H, d, *J* = 6.5 Hz, H-9). The optical rotation of 5-hydroxymellein gave [α]_D_ = −71.2° (*c* = 0.36, CH_3_OH), indicating *R*-configuration at C-3, according to the previous report [10]. These data were in agreement with those previously reported for (3*R*)-5-hydroxymellein [11].

### 2.3. Antioxidant Activities of (3R)-5-Hydroxymellein

**Figure 4:**
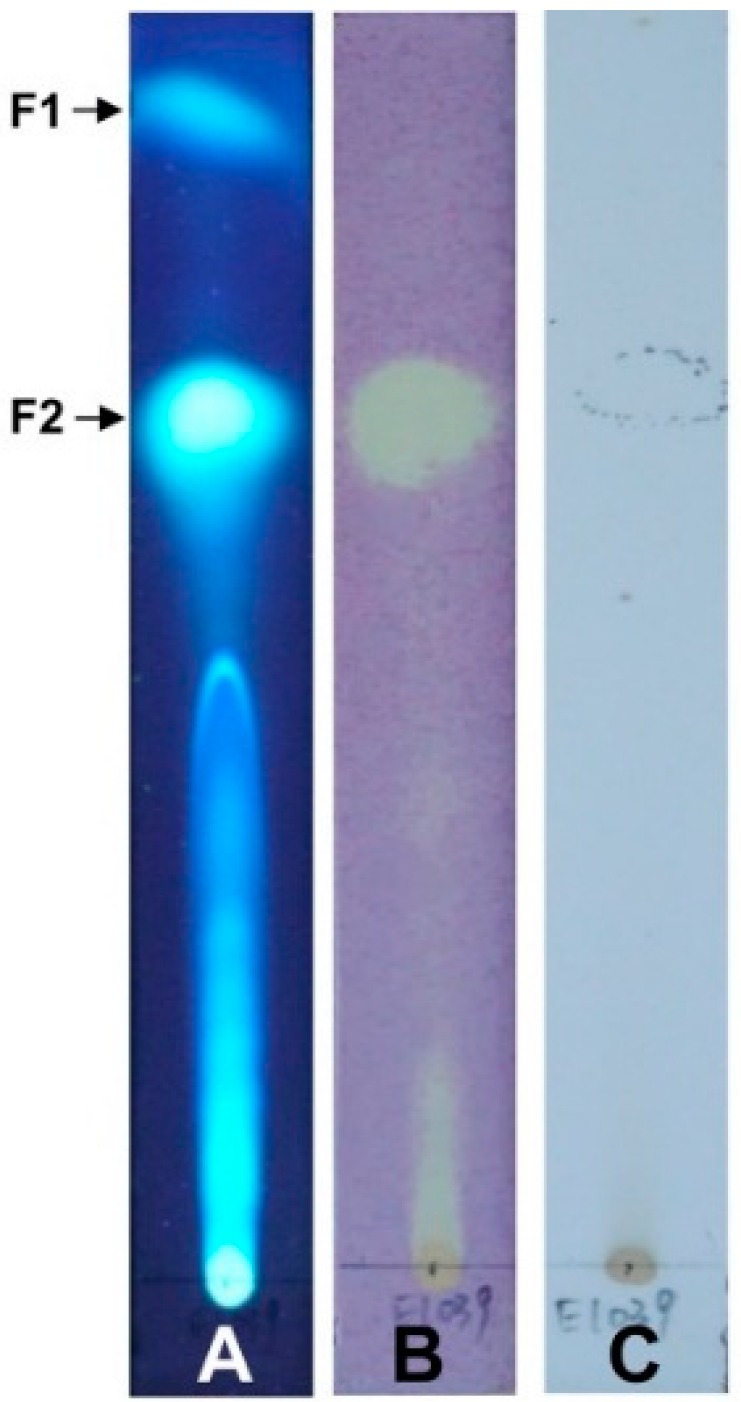
Bioautographic TLC assay of ELF000039 crude extract. (**A**) Crude extract developed on a TLC plate under UV light; (**B**) TLC plate sprayed with DPPH methanol solution under visible light; (**C**) TLC plate sprayed with 10% H_2_SO_4_ under visible light.

The results of four antioxidant activity assays are listed in Table 1. The DPPH radical is commonly used for evaluation of antioxidant activity. The DPPH scavenging capabilities of the samples were estimated by the IC_50_ values, which were 1812.8 ± 270.3 and 30.8 ± 1.4 µg/mL for crude extract and (3*R*)-5-hydroxymellein, respectively. Bioautographic TLC assay of free radical scavenging activity (Figure 4) clearly showed F2 of the chemical components in crude extract as a white yellow spot on a purple background, and that F2 underwent a relatively strong color change. As shown in Table 1, (3*R*)-5-hydroxymellein showed significantly higher DPPH radical scavenging activity than ASA (IC_50_ = 40.8 ± 2.9 µg/mL) (*p* < 0.05). The reducing power of crude extract was weak, but the IC_50_ values of (3*R*)-5-hydroxymellein and ASA were 1170.8 ± 22.1 and 1127.9 ± 12.0 µg/mL, respectively; thus, the reducing power of (3*R*)-5-hydroxymellein was considered to be close to that of ASA. The superoxide anion derived from dissolved oxygen by PMS-NADH coupling reaction reduces NBT. The decrease in absorbance at 560 nm indicated that the antioxidants consumed superoxide anions in the mixture reaction. The IC_50_ of (3*R*)-5-hydroxymellein on superoxide anion scavenging activity was 645.3 ± 5.7 µg/mL, while that of BHA was over 1000 µg/mL. Moreover, (3*R*)-5-hydroxymellein (IC_50_ = 501.8 ± 5.3 µg/mL) exhibited stronger inhibition of linoleic acid peroxidation than the positive control, ASA (IC_50_ = 755.8 ± 27.3 µg/mL).

### 2.4. Antimicrobial Activities of (3R)-5-Hydroxymellein

The antimicrobial activities of 1 mg of crude extract and (3*R*)-5-hydroxymellein against Gram-negative Gram-positive bacteria and yeast stains were tested. The data of inhibition zone diameters using disc diffusion method were shown in Table 2. Reference antibiotics did not appear inhibition zone, because 0.1 mg of cefotaxime did not reach the minimal inhibitory concentration of *Pseudomonas aeruginosa*. The discs containing 1 mg of (3*R*)-5-hydroxymellein did not form any inhibition zone against two Gram-negative bacteria (*P. aeruginosa* and *Escherichia coli*), three Gram-positive bacteria (*Staphylococcus aureus*, *Enterococcus faecium*, and *Bacillus cereus*) and one yeast stain (*Candida albicans*), which indicated (3*R*)-5-hydroxymellein had no inhibitory effect on all these six strains at the concentration of 1 mg/disc. However, ELF000039 crude extract showed partial inhibition on *E. coli* and *B. cereus*; the inhibition zone diameters were 12.2 ± 0.8 mm and 10.6 ± 0.9 mm, respectively. In a negative control, methanol had no effect on tested organisms. 

### 2.5. Cytotoxicity of (3R)-5-Hydroxymellein

The cytotoxic activities of crude extract and (3*R*)-5-hydroxymellein against the tested cell lines were determined. The crude extract exhibited low cytotoxic activities against melanoma cell lines B16F1 and B16F10, with IC_50_ values of 153.8 ± 31.1 and 221.7 ± 40.8 µg/mL, respectively. However, for HaCaT cells, the IC_50_ of crude extract was 28.6 ± 4.2 µg/mL, which indicated higher cytotoxicity than that of melanoma cells. Interestingly, (3*R*)-5-hydroxymellein showed no cytotoxic activity against B16F1, B16F10, or HaCaT cells. HaCaT cell viability was over 90% for (3*R*)-5-hydroxymellein at 100 µg/mL (Table 3).

### 2.6. Ability of (3R)-5-Hydroxymellein to Recover UVB-Induced Damage

**Figure 5:**
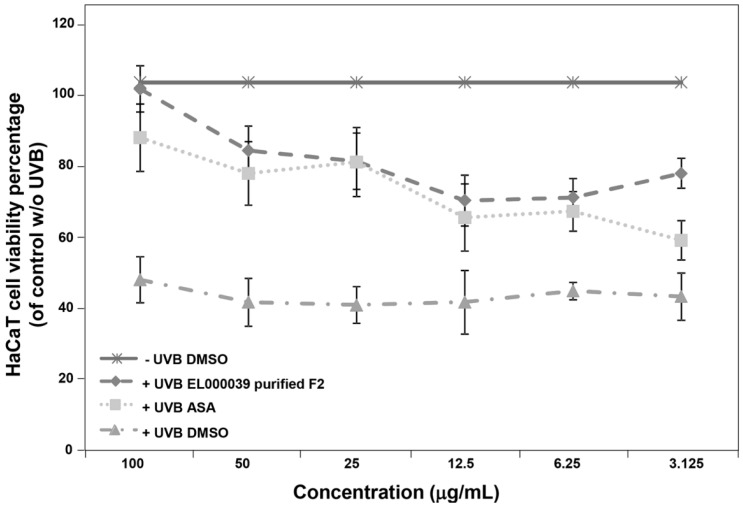
HaCaT cell viability after treatment with ELF000039 purified F2 ((3*R*)-5-hydroxymellein) and UVB irradiation. The cell survival without UVB was considered 100%; DMSO was a negative control. Data shown are the means ± SD (*n* = 3).

The ability of (3*R*)-5-hydroxymellein to recover UVB-damaged HaCaT cells is presented in Figure 5. The cell survival following UVB irradiation and DMSO (negative control) treatment ranged from 39.89% to 48.88% for all concentrations, and cell survival percentages without UVB irradiation and DMSO treatment were considered 100%. Following UVB irradiation, the viabilities of HaCaT cells treated with ASA and (3*R*)-5-hydroxymellein at 100 µg/mL concentration increased to 85% and 98%. The viabilities of HaCaT cells were higher under (3*R*)-5-hydroxymellein treatment than those of ASA, a confirmed agent for protection of skin cells from UVB [12,13], at all concentrations. These findings indicated that (3*R*)-5-hydroxymellein has a high ability to recover human keratinocytes from UVB irradiation induced damage.

### 2.7. Effect of (3R)-5-Hydroxymellein on Melanin Synthesis in B16F1 Cells

**Figure 6:**
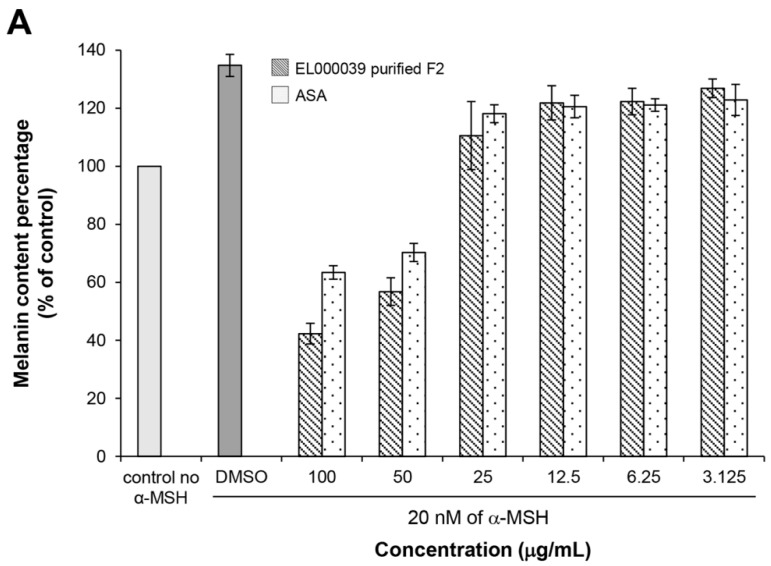

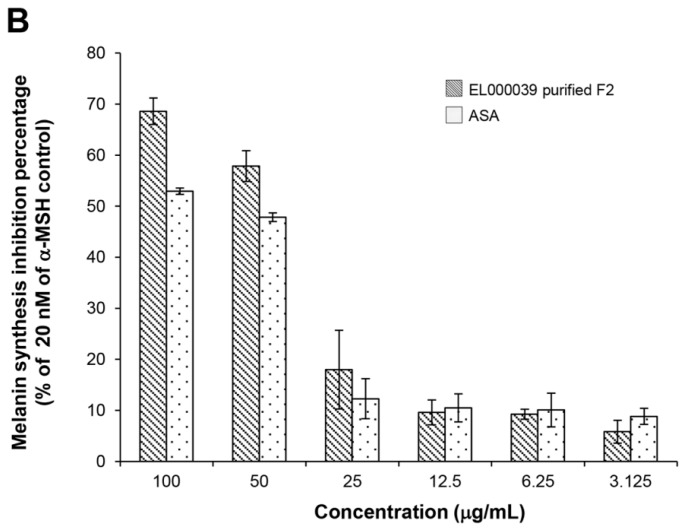
Effects of ELF000039 purified F2 ((3*R*)-5-hydroxymellein) on melanin synthesis in B16F1 cells. Cells were treated with different concentrations of (3*R*)-5-hydroxymellein or ASA under 20 nM of α-MSH. (**A**) Melanin content percentage was expressed and the melanin content of cells without α-MSH was considered 100%. DMSO was used as a negative control; (**B**) Melanin synthesis inhibition of (3*R*)-5-hydroxymellein and ASA under 20 nM of α-MSH compared to DMSO. Data shown are the means ± SD, *n* = 3.

Melanogenesis of B16F1 cells was initiated by the addition of α-melanocyte-stimulating hormone(α-MSH). The melanin content of cells treated with 20 nM of α-MSH alone increased by approximately 30% compared to no α-MSH treatment (as shown in Figure 6A). To investigate the effects of (3*R*)-5-hydroxymellein on melanogenesis, B16F1 cells were cultured in the presence of 100−3.125 µg/mL of (3*R*)-5-hydroxymellein. The melanin content compared to the control (no α-MSH) in cells was only 42.3% ± 3.6% at 100 µg/mL of (3*R*)-5-hydroxymellein. Treatment with ASA at 100 µg/mL led to an increase in cell melanin production to 63.4% ± 2.4%. The data shown in Figure 6B clearly indicate that (3*R*)-5-hydroxymellein inhibited almost 60% and 70% of melanin synthesis at 50 µg/mL and 100 µg/mL, whereas the positive control (ASA) inhibited less than 50% and 55% of melanin production, respectively, which was significantly lower than that of (3*R*)-5-hydroxymellein (*p* < 0.01). These results indicated that (3*R*)-5-hydroxymellein possessed a greater inhibitory effect on melanin synthesis by B16F1 cells than ASA at 50 and 100 µg/mL.

## 3. Discussion

(3*R*)-5-hydroxymellein was first reported as a natural product isolated from the fungal pathogen *Botryosphaeria obtuse* in 1990 [14]. Since then, the different chemical structures of 5-hydroxymellein have been found as a secondary metabolite of endophytic fungi obtained from plants several times [15], and some studies have reported that this compound has antibacterial, antifungal, and algicidal properties. For example, Bi et al. figured out S-(+)-5-hydroxymellein isolated from fungus had antibacterial activity against *Shigella sonnei*, *Mycobacterium tuberculosis*, and *Streptococcus pneumoniae* [16], while in another study, it has been proven that (3*R*)-5-hydroxymellein had antifungal, antibacterial, and algicidal properties towards *Microbotryum violaceum*, *Bacillus megaterium*, and *Chlorella fusca* [17]. However, in the present study, (3*R*)-5-hydroxymellein did not show any antimicrobial activities at a concentration of 1 mg per disc against tested microorganisms. S-(+)-5-hydroxymellein and (3*R*)-5-hydroxymellein are different configurations so that antimicrobial activities towards different bacteria strains, separately. Compared to previous studies, the tested microorganisms in the present study were different, besides, *P. aeruginosa* and *E. faecium* are recognized multidrug resistant and low antibiotic susceptibility pathogens. In addition, no studies have reported the purification of (3*R*)-5-hydroxymellein from ELF to date, despite their being important producers of secondary metabolites. Many investigations have reported that ELF produced bioactive substances, such as antioxidant activity, antimicrobial activity, and anticancer activity; however, there have been few studies of UV absorption and UV associated bioactivities.

In the present study, we purified (3*R*)-5-hydroxymellein, a natural product isolated from endolichenic fungus that absorbs UVA. Physically, the ability of a molecule to absorb light is due to electronic transitions from the highest occupied molecular orbital (HOMO) to the lowest unoccupied molecular orbital (HOMO) within the molecule. This absorbed energy can then be eliminated by heat, fluorescence, phosphorescence, or other processes to achieve photo-stability [18]. This is why (3*R*)-5-hydroxymellein showed fluorescence on the TLC plate under the UV lamp in our experiments. UV irradiation can cause photooxidative damage, especially increased generation of reactive oxygen species (ROS) in skin cells [19,20]. We demonstrated that (3*R*)-5-hydroxymellein has good antioxidant activity in vitro, with higher activity than the commercial antioxidants ASA or BHA, based on several antioxidant activity assays. Besides, a few of studies have presented that antioxidants are responsible to block UV radiation in addition to their antioxidant activities, such as ASA, carotenoids, vitamin E, and so on [21,22]. As an effective UV protectant, UV absorption activity and antioxidant activity of an agent are the most fundamental properties.

UVB radiation is currently considered to be a complete carcinogen. This is because UVB induced damage to skin cells at levels that exceed human autoimmunity increase the risk of skin diseases, including skin cancer. Thus, we examined the ability of (3*R*)-5-hydroxymellein to recover UVB induced damage in HaCaT cells before the cells UVB irradiation damage model was established. The survival of HaCaT cells treated with (3*R*)-5-hydroxymellein increased from around 50% to more than 60%, which suggested that (3*R*)-5-hydroxymellein could protect HaCaT cells from UVB irradiation. The recovery ability of (3*R*)-5-hydroxymellein toward UVB damage was consistent with the antioxidant results, with no cytotoxicity of (3*R*)-5-hydroxymellein against HaCaT cells being observed.

The cytotoxicity of (3*R*)-5-hydroxymellein against melanoma cells B16F1 and F10 was not high, indicating that 5-hydoxymellein is not a good anticancer agent. Nevertheless, it was found to inhibit melanin synthesis in B16F1 cells, and its inhibitory effects were much stronger than those of ASA; therefore, (3*R*)-5-hydroxymellein is an effective inhibitor of melanogenesis. It was inferred that the mechanisms of recovery ability toward UVB damage and melanin synthesis inhibition of (3*R*)-5-hydroxymellein occurred via the antioxidant pathway.

Many natural products and compounds have been regarded as good UV filters, for example, flavonoid extracted from high plants, mycosporine-like amino acids isolated from cyanobacteria or algae—most of which are lichen substances, such as depsidones (lobaric acid, pannarin), depsides (atranorin, gyrophoric acid), diphenylethers (epiphorellic acid), dibenzofurane derivatives (usnic acid) and bisxanthones (secalonic acid) [5,23]. Nevertheless, such photoprotective compounds are produced in limited quantities, because their sources are either long-time growth periods or small biomass, led to making their industrial application difficult. Use of ELF, which can be cultivated on a large scale, can solve this problem. Although some known fungi have been proven to be producers of UV protectants, it is easier to find novel UV absorptive and protective compounds from new species of unexplored ELF resources. Overall, the results of this study indicate that (3*R*)-5-hydroxymellein isolated from ELF was a good antioxidant with UVA absorption; it could recover UVB-induced damage and inhibit melanin synthesis. All these properties of (3*R*)-5-hydroxymellein could be considered as useful characteristics of a potential and multifunctional UV protective agent for further study.

## 4. Materials and Methods

### 4.1. Fungal Strain

The endolichenic fungus sp. ELF000039 was obtained from the Korean Lichen Research Institute (KoLRI) at Sunchon National University, Korea. ELF000039 was isolated from the lichen thalli of *Parmotrema austrosinense* (KoLRI no. 009806) collected from Jeju Island, Korea, in April 2009.

### 4.2. Fungus ITS Sequencing

The endolichenic fungus was grown and maintained on potato dextrose agar (PDA) (BD Difco, Sparks, MD, USA) at 25 °C. The total DNA of ELF was extracted using a DNeasy Plant Mini Kit according to the manufacturer’s protocols (Qiagen, Hilden, Germany). The internal transcribed spacer (ITS) region of the rDNA gene was amplified with the universal primers ITS1F (5’-CTTGGTCATTTAGAGGAAGTAA-3’) [24] and LR5 (5’-ATCCTGAGGGAAACTTC-3’) [25]. Amplifications were performed using Amplitaq DNA polymerase with buffer conditions recommended by the following parameters: initial denaturation at 94 °C for 5 min, followed by 30 cycles at 94 °C for 30 s, annealing at 55 °C for 30 s and extension at 72 °C for 30 s, then final extension at 72 °C for 10 min. The PCR product was concentrated and purified using a PCR quick-spin PCR Product Purification Kit (INTRON biotechnology, Seongnam, Korea), after which it was sequenced using the same primers.

### 4.3. Fermentation and Extraction

The fungal strain was cultured on PDA medium at 25 °C for 7 d. Mycelial agar plugs were inoculated into 500 mL Erlenmeyer flasks containing 250 mL potato dextrose broth (PDB) and incubated at 25 °C on a rotary shaker at 150 rpm for 21 d. Each culture (20 L) was then filtered to separate the filtrate and mycelia. The filtrate was extracted repeatedly with the same volume of ethyl acetate (EA), after which the organic phase was evaporated to dryness under vacuum to obtain the crude extract. Finally, the filtrate was extracted with hexane followed by EA and obtained as a brown gum that was considered the EA extract.

### 4.4. UV Spectral Scanning

The sample was diluted with ethanol and subjected to *UV* spectral analysis at 190–450 nm using a *UV*/*V*is spectrophotometer (Optizen 3220, Mecasys Co. Ltd. Daejeon, Korea).

### 4.5. Preparative Thin Layer Chromatography (TLC)

TLC analysis of crude extract was conducted in a developing system (toluene:dioxane:acetic acid = 180:45:5), after which the spots of interest were removed and extracted with acetone.

### 4.6. Substance Isolation and Identification

The EA extract (5.5 g) was separated by silica gel column chromatography (CC) using EA/hexane (1:2, *v*/*v*). Fractions were analyzed by TLC and concentrated using a rotary vacuum evaporator. Fractions (1.3 g) were then purified by silica gel column chromatography (CC) using chloroform/methanol (97:3, *v*/*v*) followed by CC over Sephadex LH20 with dichloromethane: hexane: methanol (5:5:1, *v*/*v*/*v*) to yield 40 mg of pure compound. The purity of the isolated compound was determined by HPLC analysis (LC-20A, Shimadzu, Kyoto, Japan), which was conducted under the following conditions: Column: C18 (YMC-pack ODS-A, 150 × 3.9 mm I.D); solvent system, H_2_O:Acetonitrile (3:7–7:3, gradient); flow rate: 1 mL/min; photodiode array detector (range 190–800 nm, path length: 0.5 mm, SPD-M20A, Shimadzu, Kyoto, Japan); detecting wavelength, 254 nm for HPLC and 200–800 nm for UV spectra analysis; temperature, 40 °C.

The chemical structure of the purified compound was determined by gas chromatography-mass spectrometry (GC-MS) using an Agilent 7890N GC interfaced with an Agilent 5975C mass-selective detector (70 eV, electron ionization mode) and nuclear magnetic resonance (NMR) analyses. The GC-MS system was equipped with an Ultra-2 (5% phenyl–95% methylpolysiloxane bonded phase; 25 m × 0.20 mm I.D., 0.11 μm film thickness) cross-linked capillary column (Agilent Technologies, Atlanta, GA, USA). The ^1^H-NMR spectra were acquired in 650 μL of methanol-d4 using a Bruker Avance III HD 500 MHz instrument (Bruker Biospin GmbH, Rheinstetten, Germany). Chemical shifts were calculated using tetramethylsilane (TMS) as the internal standard. The optical rotation was measured on an Autopol IV (Rudolph Research Analytical, Hackettstown, NJ, USA).

### 4.7. Bioautographic TLC Assay of Crude Extract Free Radical Scavenging Activity 

The bioautographic TLC assay was conducted according to Chaaib’s method [7]. Briefly, crude extract (10 µL, 20 mg/mL) was spotted onto the TLC plate (silica gel 60, Merck), then developed in the developing system (EA:hexane = 2:3). After developing the TLC plate, DPPH (0.5 mg/mL in methanol) was sprayed onto the plate. The active compounds were seen as yellow-white spots against a purple background. Another TLC plate was sprayed with 10% H_2_SO_4_ as a control.

### 4.8. Antioxidant Activities Assays

#### 4.8.1. DPPH Radicals Scavenging Activity

The free radical scavenging activity of samples was measured by 1, 1-diphenyl-2-picrylhydrazyl (DPPH, Sigma Aldrich, St. Louis, MO, USA) radical using a previously described method [26], with slight modification. Briefly, a reaction mixture containing 100 µL of DPPH solution (0.05 mg/mL in methanol) and 50 µL of sample solution in DMSO (1000, 500, 250, 125, 62.5 and 31.25 µg/mL) was distributed in a 96-well microplate. The mixtures were then incubated in the dark at room temperature for 30 min, after which the absorbance at 517 nm was measured in a 96-well microplate reader (Versa Max, Molecular Devices, Sunnyvale, CA, USA) against blank samples. ASA (Sigma Aldrich, USA) was used as a positive control. Each concentration and all tests were conducted in triplicate and the results were averaged.

DPPH scavenging activity was calculated using the following equation: DPPH scavenging activity (%) = [1 − (A_sample_ − A_blank_)/A_control_] × 100%, where the A_sample_ was the absorbance of the reaction mixture or standards and A_control_ was the absorbance of the negative control.

The inhibition concentration at 50% (IC_50_) was used to compare the scavenging activity. This value was calculated based on regression analyses using the SPSS 17.0 software (IBM, Armonk, NY, USA).

#### 4.8.2. Reducing Power

Reducing power was determined as previously described [26,27], with slight modification. Briefly, 100 µL of sample (1000, 500, 250, 125, 62.5, and 31.25 µg/mL) was mixed with 250 µL of phosphate buffer (0.2 M, pH 6.6) and 250 µL of potassium ferricyanide (1%), then incubated at 50 °C for 20 min. After being allowed to cool, 250 µL of trichloroacetic acid (10%) was added to the reaction mixture, which was then centrifuged at 3000 rpm for 10 min. Finally, 250 µL of the upper layer of solution was mixed with 250 µL of distilled water and 50 µL of ferric chloride (6 mM) and the absorbance of 150 µL of the mixture was measured at 700 nm in a 96-well microplate reader. A higher absorbance of the mixture indicated that the reducing power had increased. ASA was used as a positive control. The capacity to reduce Fe^3+^ was expressed as IC_50_, corresponding to the effective concentration at which the absorbance was equal to 0.5.

#### 4.8.3. Superoxide Anion Radical Scavenging Activity

The superoxide anion radical scavenging activity of samples was measured as previously described [28], after modification for microplates. Briefly, 150 µL of nitroblue tetrazolium (NBT, Sigma Aldrich, USA) solution (156 µM in 0.1 M phosphate buffer, pH 7.4), 150 µL of NADH (Sigma Aldrich, USA) solution (468 µM in 0.1 M phosphate buffer, pH 7.4), and a 75 µL of sample (1000, 500, 250, 125, 62.5, and 31.25 µg/mL) were mixed. The reaction was then started by adding 15 µL of phenazine methosulphate (PMS, Sigma Aldrich, USA) solution (60 µM PMS in 0.1 M phosphate buffer, pH 7.4), after which the mixture was incubated at room temperature for 10 min. The absorbance of 150 µL of the mixture was then measured at 560 nm in a 96-well microplate reader. BHA was used as a positive control.

The inhibition of superoxide anion generation was calculated using the following equation: superoxide anion scavenging activity (%) = [1 − (A_sample_ − A_blank_)/A_control_] × 100%, where A_sample_ was the absorbance of the reaction mixture or standards and A_control_ was the absorbance of the negative control. The IC_50_ was calculated using the SPSS 17.0 software (regression analyses).

#### 4.8.4. Linoleic Acid Assay

The inhibition of linoleic acid peroxidation of samples was measured according to the ferric thiocyanate method [29,30], with some modifications. Briefly, 50 µL of sample (1000, 500, 250, 125, 62.5 and 31.25 µg/mL) was added to 100 µL of linoleic acid emulsion (0.02 M, pH 7.0) and 100 µL of phosphate buffer (0.2 M, pH 7.0). The mixture was then incubated at 37 °C for 24 h. Previously, 0.02 M linoleic acid emulsion was prepared by mixing 0.2804 g of linoleic acid (Sigma Aldrich, USA) with the same weight of Tween 20 and a 50 mL phosphate buffer (0.2 M, pH 7.0), after which the mixture was homogenized. Following incubation, 1 mL of ammonium thiocyanate (3%, *w*/*v*) and 100 µL of FeCl_2_ (0.02 M in 1 M HCl) were added to 100 µL of the mixture. The inhibition activity was then determined based on the absorbance at 500 nm in a spectrophotometer. ASA was used as a positive control and the solution without the samples was used as a negative control.

The inhibition of linoleic acid peroxidation was calculated as follows: inhibition percentage (%) = [1 − (A_sample_ − A_blank_)/A_control_] × 100%, where A_sample_ was the absorbance of reaction mixture or standards and A_control_ was the absorbance of the negative control. The IC_50_ was calculated using the SPSS 17.0 software (regression analyses).

### 4.9. Antimicrobial Activity Assay

#### 4.9.1. Microorganisms and Media

The following bacteria were used as test organisms in this study: two Gram-negative stains: *Pseudomonas aeruginosa* (CCARM 2202) and *Escherichia coli* (ATCC 8739); three Gram-positive stains: *Staphylococcus aureus* (CCARM 3A048), *Enterococcus faecium* (CCARM 5200), and *Bacillus cereus* (ATCC 11778). *P. aeruginosa*, *S. aureus*, and *E. faecium* were maintained on brain heart infusion (BHI) broth or agar medium (BD Difco, Sparks, MD, USA). *E. coli* and *B. cereus* were cultured on nutrient agar medium. The yeast used as test organism was *Candida albicans* (ACTT 11006) grown on yeast mold medium. All the microorganisms were obtained from Culture Collection of Antimicrobial Resistant Microbes (CCARM) and American Type Culture Collection (ATCC).

#### 4.9.2. Antimicrobial Activity Assay Using the Disc Diffusion Method

Disc diffusion method for antimicrobial activity testing was carried out according to the standard method by Bauer et al. [31] and Zaidan et al. [32]. Briefly, all the test samples were dissolved completely in methanol in order to obtain concentrations of 10 mg/mL. Inoculum of bacterial and yeast strains (10^8^ CFU/mL) were swabbed on the corresponding agar plate in a petri dish (90 mm) using sterile swab. The 8 mm sterile paper disc (ADVANTEC, Kanagawa-Ken, Japan) was impregnated with 100 µL of samples (1 mg). These discs were gently pressed in corresponding agar plates and incubated at 37 °C for 24 h. The disc with methanol was used as negative control. In addition, cefotaxime, vancomycin, and ketoconazole (Sigma Aldrich, USA) were used as reference antibiotics and concentrations were 1 mg/mL. The antimicrobial activity was determined by measuring inhibition zone diameters (mm). All experiments were performed in triplicate.

### 4.10. Cell Experiments

#### 4.10.1. Cell Lines and Culture Conditions

The B16F1 and B16F10 mouse melanoma cell lines were kindly provided by Professor Hangun Kim, College of Pharmacy, Sunchon National University. The cells were cultured in RPMI-1640 culture medium (GenDEOPT, Katy, TX, USA) supplemented with 10% fetal bovine serum (FBS, GenDEOPT, USA) and 1% penicillin-streptomycin (P/S) solution (100 IU penicillin and 100 µg/mL streptomycin) in a humidified atmosphere containing 5% CO_2_ at 37 °C. HaCaT cells, which are immortalized human keratinocytes, were a gift from Professor Seonggene Lee, Chonnam National Univerisity. HaCaT cells were maintained in DMEM medium containing 10% FBS and 1% P/S solution in a humidified 5% CO_2_ atmosphere at 37 °C.

#### 4.10.2. Cytotoxicity assay

##### Treatment of Cell Lines

Stock solutions (10 mg/mL) of all samples dissolved in DMSO were diluted in corresponding culture medium to the required working concentrations. B16F1 and B16F10 cells (3000 cells/well) and HaCaT cells (5000 cell/well) were seeded into 96-well microplates. After cells were allowed to adhere for 24 h, different concentrations (final concentrations 100, 50, 25, 12.5, 6.25 and 3.125 µg/mL) of samples were added to the wells, except for the control wells, which contained only culture medium and DMSO. All samples were then incubated for 48 h in a humidified 5% CO_2_ atmosphere at 37 °C, and all treatments were conducted in triplicate.

##### Cell Viability Assay (MTT Test)

The relative viable cell number was determined by the MTT method. In brief, the treated cells were incubated for 4 h in fresh culture medium containing 0.5 mg/mL of 3-(4,5-dimethylthiazol-2-yl)-2,5-diphenyl-tetrazolium bromide (MTT, Sigma Aldrich, USA) solution at 37 °C in a humidified 5% CO_2_ atmosphere. At the end of this period, the supernatant was discarded and replaced with DMSO, and the absorbance of dissolved formazan was recorded at 540 nm using a microplate reader. The cell viability in control wells was considered to be 100%, and the IC_50_ concentration was defined as the concentration of sample inhibiting the cell viability by 50%.

#### 4.10.3. Cell UVB Irradiation

Cells were irradiated using an in vitro irradiation model [33,34], with modifications to investigate the ability to recover UVB induced damage. The HaCaT cells (6000 cells/well) were seeded into 96-well microplates and incubated in a humidified atmosphere containing 5% CO_2_ at 37 °C. After 24 h, the medium of the cells attached to microplates was replaced with phosphate buffer saline (PBS, pH 7.4). PBS was used during the UV irradiation period because culture medium contains radical scavengers or UV absorbing substances such as phenol red and proteins [35]. The near confluence cells were plated at 15 cm from a UVB lamp (T-15M, Vilber-Lourmat, France) and exposed to UV irradiation for 45 s, while another plate was kept in the darkness at room temperature for as long as the irradiation period. These irradiation conditions (15 cm distance, 45 s) were necessary to reach approximately 50% cell survival without any UV protectors. The irradiated or unirradiated HaCaT cells were then incubated in fresh culture medium containing different concentrations (final concentrations of 100, 50, 25, 12.5, 6.25 and 3.125 µg/mL) of samples for 4 h at 37 °C in a CO_2_ incubator. Finally, the relative viable cell number was determined by the MTT method. The viability of cells without UV irradiation and samples were considered to be 100%. ASA was used as a positive control. DMSO was the negative control.

#### 4.10.4. Melanin Content Assay

Melanin content was estimated according to a modified version of the method described by Hosoi et al. [36]. Briefly, B16F1 cells (3 × 10^4^ cell/well) were cultured with 20 nM of α-MSH (Sigma Aldrich, USA) in a 6-well plate. After 24 h of incubation, the medium was changed to one containing the different concentrations of samples (final concentrations were 100, 50, 25, 12.5, 6.25 and 3.125 µg/mL) for 72 h. After washing with PBS, the cells were harvested by trypsinization and the number of viable cells was counted by the trypan blue exclusion method. The cell pellet was solubilized in 1 N NaOH containing 10% DMSO at 80 °C for 30 min, after which the amount of melanin was determined based on the absorbance at 475 nm using a spectrophotometer and the results were expressed as the melanin content per cell. ASA was used as a positive control. DMSO was the negative control.

## 5. Conclusions

In this study, (3*R*)-5-hydroxymellein was purified from secondary metabolites of endolichenic fungus. The results revealed that (3*R*)-5-hydroxymellein was a highly effective antioxidant possessing UVA absorption, non-cytotoxic against B16F1, B16F10 and HaCaT cell lines. Moreover, (3*R*)-5-hydroxymellein could protect HaCaT cells from UVB irradiation and inhibit melanin synthesis in B16F1 cells. Taken together, these results suggest that (3*R*)-5-hydroxymellein can have an interesting and promising profile to go further in development as a multifunctional and potential skin UV protectant.

## Figures and Tables

**Table 1:** Antioxidant activities (IC_50_; μg/mL) of ELF000039 crude extract, purified F2 ((3*R*)-5-hydroxymellein), and positive controls (ASA and BHA).

Compound	DPPH Scavenging Activity	Reducing Power	Superoxide Anion Scavenging Activity	Inhibition of Linoleic Acid Peroxidation
Crude extract	1812.8 ± 270.3	ND ^a^	>2000	>2000
(3*R*)-5-hydroxymellein	30.8 ± 1.4	1170.8 ± 22.1	645.3 ± 5.7	501.8 ± 5.3
ASA	40.8 ± 2.9	1127.9 ± 12.0	-	755.8 ± 27.3
BHA	- ^b^	-	>1000	-

^a^ no data; ^b^ not tested; data are the means ± SD (*n* = 3).

**Table 2:** Antimicrobial activities (inhibition zone diameters, mm) of ELF000039 crude extract, purified F2 ((3*R*)-5-hydroxymellein), and reference antibiotics against microorganisms.

Compound	Microorganism					
	*P. aeruginosa*	*E. coli*	*S. aureus*	*E. faecium*	*B. cereus*	*C. albicans*
Crude extract (1 mg)	0	PI 12.2 ± 0.8	0	0	10.6 ± 0.9	0
(3*R*)-5-hydroxymellein (1 mg)	0	0	0	0	0	0
Vancomycin (0.1 mg)	-	- ^a^	12.5 ± 0.9	16.9 ± 1.4	16.4 ± 0.9	-
Cefotaxime (0.1 mg)	0	28.3 ± 1.1	-	-	-	-
Ketoconazole (0.1 mg)	-	-	-	-	-	39.8 ± 0.4

^a^ not tested; data are the means ± SD (*n* = 3).

**Table 3:** Viabilities of three cell lines treated with different concentrations of (3R)-5-hydroxymellein.

Cell Line	Concentration (µg/mL)				
	100	50	25	12.5	6.25
B16F1	92.2 ± 10.6	105.3 ± 1.3	109.8 ± 4.9	100.5 ± 0.7	99.9 ± 5.3
B16F10	99.6 ± 10.1	114.6 ± 4.3	109.9 ± 9.5	110.0 ± 6.2	103.0 ± 3.6
HaCaT	96.5 ± 5.8	94.5 ± 4.3	101.1 ± 5.1	116.8 ± 13.1	89.3 ± 8.1

Data are the means ± SD (*n* = 3).
